# A proteomic analysis of seeds from *Bt*-transgenic *Brassica napus* and hybrids with wild *B. juncea*

**DOI:** 10.1038/srep15480

**Published:** 2015-10-21

**Authors:** Yongbo Liu, Ying-Xue Zhang, Song-Quan Song, Junsheng Li, C. Neal Stewart Jr., Wei Wei, Yujie Zhao, Wei-Qing Wang

**Affiliations:** 1 State Key Laboratory of Environmental Criteria and Risk Assessment, Chinese Research Academy of Environmental Sciences, 8 Dayangfang, Beijing 100012, China; 2 Key Laboratory of Plant Resources and Beijing Botanical Garden, Institute of Botany, Chinese Academy of Sciences, Beijing 100093, China; 3 State Key Laboratory of Vegetation and Environmental Change, Institute of Botany, Chinese Academy of Sciences, Beijing 100093, China; 4 College of Chemistry and Chemical Engineering, Henan University, Kaifeng, 475001, China; 5 Department of Plant Sciences, University of Tennessee, 2431 Joe Johnson Drive, Knoxville, TN 37996-4561, USA

## Abstract

Transgene insertions might have unintended side effects on the transgenic host, both crop and hybrids with wild relatives that harbor transgenes. We employed proteomic approaches to assess protein abundance changes in seeds from *Bt*-transgenic oilseed rape (*Brassica napus*) and its hybrids with wild mustard (*B. juncea*). A total of 24, 15 and 34 protein spots matching to 23, 13 and 31 unique genes were identified that changed at least 1.5 fold (p < 0.05, Student’s t-test) in abundance between transgenic (tBN) and non-transgenic (BN) oilseed rape, between hybrids of *B. juncea* (BJ) × tBN (BJtBN) and BJ × BN (BJBN) and between BJBN and BJ, respectively. Eight proteins had higher abundance in tBN than in BN. None of these proteins was toxic or nutritionally harmful to human health, which is not surprising since the seeds are not known to produce toxic proteins. Protein spots varying in abundance between BJtBN and BJBN seeds were the same or homologous to those in the respective parents. None of the differentially-accumulated proteins between BJtBN and BJBN were identical to those between tBN and BN. Results indicated that unintended effects resulted from transgene flow fell within the range of natural variability of hybridization and those found in the native host proteomes.

Commercial release of genetically modified (GM) crops has led to various discussions of unintended effects. One source of potential unintended effects is the random insertion of transgenes in plant genomes that might lead to inadvertent genomic alterations (e.g. deletions, insertions, and rearrangements), biochemical modification, or other secondary or pleiotropic effects^1^,^2^,^3^,^4^,^5^. Intended and unintended alterations might change plant-derived products^6^, which could affect “substantial equivalence” of the GM crops and derived feed and food compared with those that are accepted as safe^7^. Assessment of substantial equivalence typically focuses on well-known toxic or nutritionally harmful outcomes, such as allergenicity^7^, but unknown unintended side effects are typically less clear with regards to discovery and characterization. In recent years, ‘omic’ approaches have been used to analyze the entire composition of classes of compounds in organisms, including genomics (all genes), transcriptomics (all expressed genes), metabolomics (all metabolites), and proteomics (all proteins). These have all been used to characterize GM crops^5^,^8^,^9^,^10^,^11^,^12^,^13^. These approaches allow, in theory, a holistic search for unintended alterations in GM plants^5^.

Proteomic analysis might be especially useful in biosafety assessments of GM crops since proteins are gene products responsible for much of plant metabolism and growth. Proteins are important components of cytoskeletons, membranes and cell walls. Moreover, some proteins are toxic, antinutritional, or allergenic, which could have negative impact on human health. Proteomics analyses have been applied to test for unintended effects in GM crops, such as tomato^6^,^10^, rice^11^,^14^, maize^8^,^15^,^16^, wheat^17^, pea^18^,^19^ and tobacco^20^. Most of these studies showed that transgenic lines did have some changes in the production of proteins—those that were not targets for genetic engineering^15^,^16^,^17^,^20^. An important question to ask is how altered protein production compares with the range of natural variability. Assessing the proteomics of seeds is especially appropriate for edible seed crops, and relatively facile given the relative compact nature of the seed proteome.

In certain hosts, transgenes could be transferred into hybrids through outcrossing of GM crops with crop varieties or wild relatives, and in some cases, introgressed transgenic advanced generations could occur^21^,^22^,^23^,^24^,^25^. Gene flow from GM crops to their wild relatives is one main environmental regulatory issue^22^,^24^,^26^, with the concern of increasing risks, such as increased weediness^27^. In examples of transgenes conferring increased insect, herbicide or virus-resistance, there might be, in turn, a competitive advantage of GM plants when cultivated with non-GM plants^26^,^28^. Previous studies of transgene flow have focused on effects on plant phenotypic and agronomic characteristics, plant fitness and ecological risks^29^,^30^,^31^,^32^.

Oilseed rape (*Brassica napus*) has been a widely-used crop to study the ecological consequence of gene flow^25^. Wild brown mustard (*B. juncea*), an allotetraploid wild relative species of *B. napus*, is a widespread weed in agricultural fields in China and elsewhere. Hybrids between *B. napus* and wild *B. juncea* are successfully obtained by open pollination^21^,^30^. The aim of this study was, for the first time, to perform a proteomic study in a transgenic *B. napus* and *B. napus*/*B. juncea* hybrid system to better understand potential unintended effects of GM event.

## Results

**Figure 1:**
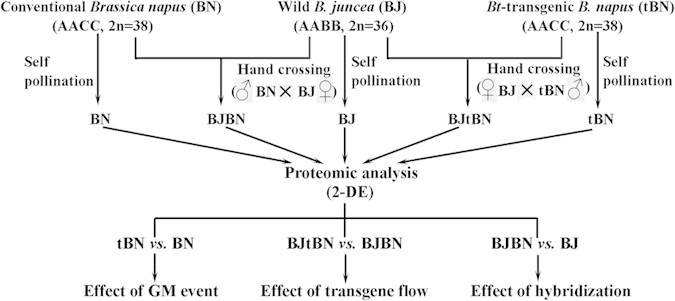
Scheme of proteomic evaluation of transgenic effects on *Brassica napus* and its hybrid with wild *B. juncea*. Wild *B. juncea* (BJ) was hybridized with transgenic (tBN) or conventional (BN) *B. napus* by hand crossing to form F1 hybrids, BJtBN and BJBN, respectively. At the same time, BN, tBN and BJ plants were self-pollinated. tBN, BN, BJ, BJtBN and BJBN seeds were collected separately after maturation and then used for proteomic analysis by 2-D electrophoresis (2-DE).

We compared proteomes between conventional (BN) and transgenic (tBN) *B. napus* seed or among wild *B. juncea* (BJ) and their hybrid BJ × BN (BJBN) and BJ × tBN (BJtBN) seeds by the 2-D electrophoresis (2-DE, Fig. 1). Proteomic comparison between tBN and BN seeds detected the potential unintended effects of GM event. By comparing BJtBN versus BJBN seed proteomes, it is possible to evaluate the unintended influences by transgene flow. At the same time, the comparison of BJBN versus BJ seed proteomes could investigate the natural variability of hybridization (Fig. 1).

### Proteomic analysis of GM event effects: tBN vs. BN

**Figure 2:**
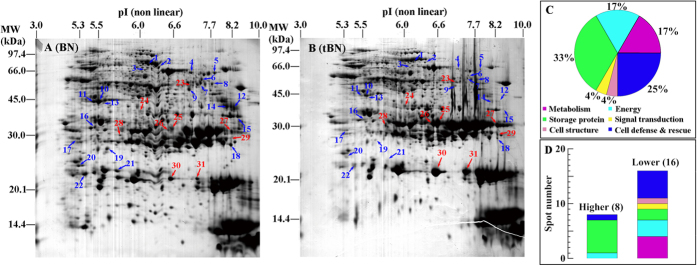
Proteomic comparison of non-transgenic (BN) and transgenic *B napus* (tBN) seeds. (**A**,**B**) representative CBB R-250 stained gels of BN and tBN seeds, respectively. A total of 400 μg of proteins was loaded onto the gel strip and separated by 2-D gel electrophoresis and visualized with CBB R-250. The red and blue arrows with numbers indicate the spots whose abundance were more than 1.5 fold higher and lower in tBN than in BN seeds, respectively. The spots were excised, and proteins were extracted and finally identified by MS/MS. Only protein spots successfully identified by MS/MS and matched to one protein were used in the functional analysis (**C**,**D**). (**C**) functional distribution of identified differentially accumulated protein spots between tBN and BN seeds; (**D**) number and distribution of identified protein spots accumulated in higher or lower abundance in tBN than in BN seeds.

Approximately 800 protein spots were detected in 2-D gels of tBN and BN seeds after Coomassie brilliant blue R-250 (CBB) staining (representative image in Fig. 2A,B). We considered the proteomic differences between tBN and BN seeds significant if they had greater than a 1.5-fold change in abundance at a P-level of p < 0.05 using Student’s t-test, which resulted in a total of 31 protein spots meeting these criteria (Fig. 2A,B, Supplemental Table S1). Proteins were successfully identified in 28 spots by searching the PMFs of each protein spot against the NCBInr database. Among these spots, 24 were comprised of just one protein, which could be assigned to 23 respective unique genes (Table 1). The other four genes were matched to two proteins (Supplemental Table S2). If one spot consisted of two proteins, it is difficult to deconvolute protein identity. Therefore, the only spots that were analyzed herein are those with a one-to-one match.

The proteins were classified into six functional categories according to Bevan *et al.* (1998)^33^ (Table 1) and mainly involved in four functional categories, storage proteins (33%), cell defense and rescue (25%), energy (17%), and metabolism (17%) (Fig. 2C). Eight spots accumulated in higher abundance in tBN than in BN seeds (Fig. 2D, Table 1). Among these spots, six were storage proteins, and the other two involved in energy production and cell defense and rescue (Fig. 2D, Table 1). The other 16 protein spots were accumulated in lower abundance in tBN than in BN seeds (Fig. 2D, Table 1). Five of them involved in cell defense and rescue, four in metabolism and three in energy production (Fig. 2D, Table 1). Four protein spots, BnaC06g06810D (spot 12), Cruciferin storage protein (spot 8), BnaC03g41580D (spot 15) and BnaA08g25110D (spot 16), were found in BN but not in tBN (Table 1). No protein products derived from the *Bt Cry1Ac* and *gfp* transgenes were identified in the proteomic analysis.

### Proteomic analysis of transgene flow effects: BJtBN vs. BJBN

**Figure 3:**
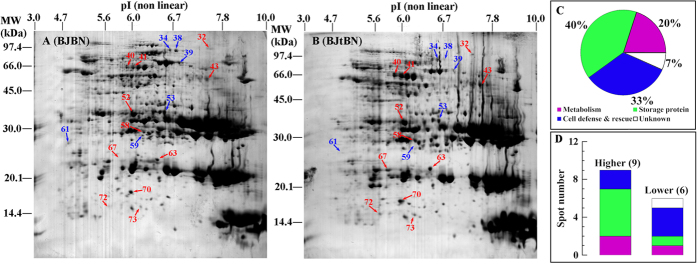
Proteomic comparison of BJtBN and BJBN hybrid seeds. BJBN, hybrids of *B juncea* (BJ) with non-transgenic *B. napus*; BJtBN, hybrids of BJ with transgenic *B napus*. (**A**,**B**) representative CBB R-250 stained gels of BJBN and BJtBN seeds, respectively. A total of 400 μg of proteins was loaded onto the gel strip and separated by 2-D gel electrophoresis and visualized with CBB R-250. The red and blue arrows with numbers indicate the spots whose abundance were more than 1.5 fold higher and lower in BJtBN than in BJBN seeds, respectively. The spots were excised, and proteins were extracted and finally identified by MS/MS. Only protein spots successfully identified by MS/MS and matched to one protein were used in the functional analysis (**C**,**D**). (**C**) functional distribution of identified differentially accumulated protein spots between BJtBN and BJBN seeds; (**D**) number of identified protein spots accumulated in higher or lower abundance in BJtBN than in BJBN seeds.

A total of 17 protein spots were found to vary more than 1.5 fold (p < 0.05) in abundance between BJtBN and BJBN seeds (Fig. 3A,B, Supplemental Table S3). Fifteen protein spots were successfully identified and all matched to only one protein (Table 2). These proteins belonged to 13 unique genes (Table 2).

These identified proteins were mainly involved in three categories, storage protein (40%), cell defense and rescue (33%), metablolism (20%) (Fig. 3C). Nine protein spots accumulated higher in BJtBN than in BJBN seeds, and six had lower abundance in BJtBN (Fig. 3D, Table 2). Among the spots with higher abundance in BJtBN seeds, five were storage proteins, two were involved in cell defense and rescue, and the remaining two had functions related to metabolic processes (Fig. 3D, Table 2). Two protein spots, cruciferin storage protein (spot 72) and BnaC03g55840D (spot 67), were found in BJtBN but not in BJBN seeds (Table 2). For those with lower abundance, three had functions in cell defense and rescue, one was a storage protein, and one each had metabolic and unknown functions (Fig. 3D, Table 2).

### Proteomic analysis of hybridization effects: BJBN vs. BJ

**Figure 4:**
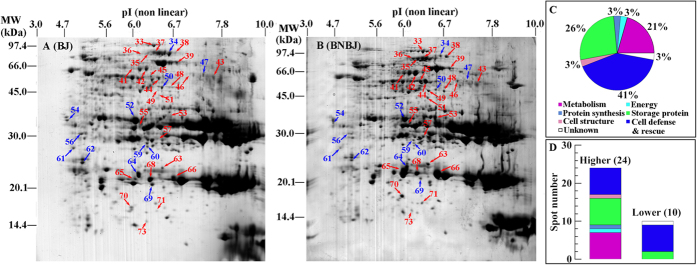
Proteomic comparison of BJ and BJBN seeds. BJ, wild *B juncea*; BJBN, hybrid of BJ with non-transgenic *B. napus*. (**A**,**B**) representative CBB R-250 stained gels of BJ and BJBN seeds, respectively. A total of 400 μg of proteins was loaded onto the gel strip and separated by 2-D gel electrophoresis and visualized with CBB R-250. The red and blue arrows with numbers indicate the spots whose abundance were more than 1.5 fold higher and lower in BJBN than in BJ seeds, respectively. The spots were excised, and proteins were extracted and finally identified by MS/MS. Only protein spots successfully identified by MS/MS and matched to one protein were used in the functional analysis (**C**,**D**). (**C**) functional distribution of identified differentially accumulated protein spots between BJBN and BJ seeds; (**D**) number of identified protein spots accumulated in higher or lower abundance in BJBN than in BJ seeds.

Thirty-seven protein spots changed more than 1.5-fold in abundance in hybrid BJBN seeds compared to the seeds of its the maternal BJ parent (Fig. 4A,B, Supplemental Table S4). Proteins were identified in 35 spots, of which 34 spots matched to only one protein and derived from 31 unique genes (Table 3). One spot (spot 56) matched to two proteins (Supplemental Table S2) and was excluded in the following analysis.

The identified protein spots that varied in abundance between BJBN and BJ were annotated to cell defense and rescue (41%), storage protein (26%) and metabolic functions (21%) (Fig. 4C). Twenty-four spots, involved mainly in cell defense and rescue (seven spots), metabolism (seven spots) and storage (seven spots), had higher abundance in BJBN than in BJ seeds (Fig. 4D, Table 3). The abundance of the other ten protein spots was lower in BJBN than in BJ seeds, among which, cell defense and rescue (seven spots) were the most abundant (Fig. 4D, Table 3).

### Comparison of proteome changes associated with GM event, transgene flow and hybridization

**Figure 5:**
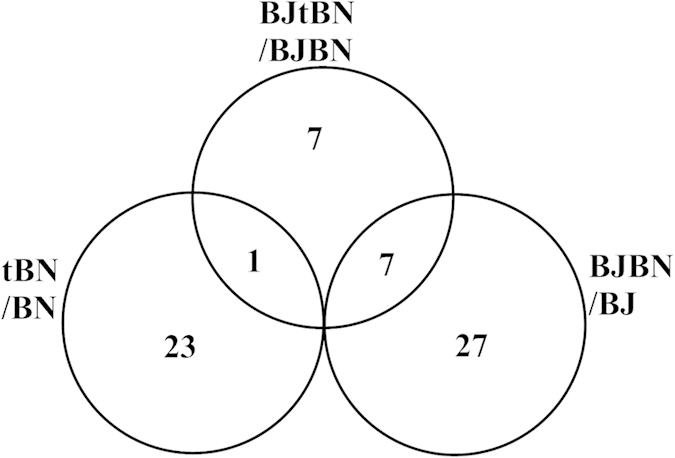
Venn diagram of differentially accumulated protein spots as consequences of three comparisons. tBN/BN, proteomic comparison of transgenic (tBN) and non-transgenic *B napus* (BN) seeds for detecting GM event; BJtBN/BJBN, proteomic comparison of hybrids *B juncea* (BJ) × tBN (BJtBN) and BJ × BN (BJBN) seeds for transgene flow; BJBN/BJ, proteomic comparison of BNBJ and BJ seeds for hybridization.

One protein, BnaC01g09900D exhibited a higher accumulation in tBN than in BN seeds (spot 26) and in BJtBN than in BJBN seeds (spot 32) (Fig. 5; Tables 1 and 2). However, this protein accumulated in spots 26 and 32 had completely different experimental molecular mass and pI (Tables 1 and 2), indicating that they have been differentially modified after translation. Therefore, none of protein spots accumulated differentially in abundance between BJtBN and BJBN seeds were substantially identical to those between tBN and BN seeds.

Seven protein spots (spot 41, 43, 73, 70, 34, 61, 59) varied similarly in abundance between BJtBN/BJBN and BJBN/BJ comparisons (Fig. 5; Tables 2 and 3). No identical proteins were found between pair-wise comparisons of tBN *vs.* BN and BJBN *vs.* BJ (Fig. 5; Tables 1 and 3).

## Discussion

### Proteomic comparison between tBN and BN seeds did not detect new known toxic or nutritionally-harmful compounds

Twenty four proteins were identified to change in abundance in oilseed rape as a result of transgenic events (Fig. 2, Table 1). This number fell within the range of previous proteomic studies on seeds of transgenic crops. A proteomic analysis revealed that 43 protein spots increased or decreased in abundance in GM maize seeds compared with its non-GM isoline^16^. Gong *et al.* (2012) found that 17 protein spots varied in abundance between insect-resistant transgenic rice harboring *Cry1Ac* and its non-transgenic control, and 12 spots between herbicide-resistant transgenic rice carrying *bar* and non-transgenic control^11^. A proteomic study on transgenic tomato reported that transgene insertion had no influence on seed proteomes^10^.

To date no proteomic studies on transgenic plants have raised any new safety concerns^3^,^5^; our study did not change this paradigm. In the seeds of oilseed rape, no new protein was found in tBN seeds when compared to BN seeds (Table 1). Abundance of eight protein spots was higher in tBN than in BN seeds (Fig. 2D). They are cruciferin gene family proteins (spots 25, 26, 27, 29, 30 and 31), glyceraldehyde-3-phosphate dehydrogenase (spot 24) and catalase (spot 23) (Table 1). Cruciferin is one type of storage proteins in oilseed rape seeds. Storage proteins are importantly nutritional compounds in seeds and play essential roles in seed germination and seedling growth^34^,^35^,^36^. Glyceraldehyde-3-phosphate dehydrogenase is a well-known glycolytic enzyme, and catalase is one of the antioxidant enzymes for removal of reactive oxygen species (ROS)^37^,^38^. None of these proteins has been reported to be toxic or nutritionally harmful to human health. The result is what was expected, since these two *Brassica* species are not known to produce toxic proteins in seeds. Nonetheless, our study was about unintended effects, and it is conceivable that there could have been novel protein biosynthesis in the seeds; we found none. Regardless, the spontaneous production of novel allergens and toxins in transgenic is a long-held point of concern by members of the public.

Accumulation of several protein spots in BN seeds were completely inhibited after transgenic modification (Fig. 2A,B, Table S1). This phenomenon can also be found in other proteomic evaluation of GM events^16^,^18^,^19^. It may be due to the random insertion of transgene into plant genome, which inhibits the gene expression and accumulation of these proteins.

In seeds, it appears that storage protein is most susceptible to be altered by transgene insertion. Storage proteins are the common proteins found to vary in abundance between GM and non-GM seeds in many species, such as rice^11^, maize^16^ and pea^18^,^19^. In oilseed rape, we found that about 33% of the differentially accumulated proteins in tBN seeds were storage proteins when compared to BN seeds (Fig. 2C). Transgenic events seemed to influence not only storage protein biosynthesis, but also their post-translational modification. In oilseed rape seeds, storage proteins varying in abundance between transgenic and non-transgenic seeds had different experimental molecular weights (MW), pI, or both, respective to their theoretical MW and pI (Table 1). Similar results have also been found in proteomic studies in seeds of transgenic maize^16^ and pea^18^,^19^. Storage proteins are the most abundant in seeds and account for as much as 60% of total proteins^39^. This finding suggests that storage proteins might be among the most susceptible proteins for alteration in transgenic plants.

Besides storage proteins, some other proteins accumulated differentially in abundance between tBN and BN also exhibited different experimental MW and pI as compared with theoretical MW and pI (Table 1). Similar results were also found in the comparison of BJtBN and BJBN seeds (Table 2) and in previous proteomic studies on GM plants^9^,^11^,^16^,^18^. These results suggest that a GM event might affect both protein synthesis and post-translational modification.

No protein products derived from the inserted transgenes (*Cry1Ac* and *gfp*) were identified in the present proteomic analysis. Similar results have also been found in other proteomic studies on transgenic crops^11^,^15^,^16^,^40^. This may be due to that expression of transgene protein products in tBN seeds is under the sensitivity of CBB staining for detection of protein spots in 2-D gel. In addition, these and other low-produced protein would be occluded by highly synthesized storage proteins, which dominated the 2-D gel (for example, Fig. 2A,B, in area at about 30 kDa, pI 6.6–8.2, 20.1 and 14.4 kDa, pI 7.7–10).

### Proteomic changes in transgenic hybrids were within the bounds of natural variability

As in tBN seeds, proteomics analysis found no transgene protein products in BJtBN seeds. However, a Bt ELISA detected accumulation of Bt-toxin proteins in the BJtBN seeds (Supplemental Figure S1), showing gene flow to the wild relative^41^,^42^. Transgene flow has potential effects on plant genome, morphology traits and plant growth and reproduction, which may confer altered host ecology^22^,^26^,^27^.

Transgene flow is likely to result in unintended effects in transgenic hybrids. Compared with non-transgenic hybrid seeds of BJBN, fifteen protein spots varied in abundance in the transgenic hybrid seeds of BJtBN (Fig. 3A,C, Table 2). BJBN and BJtBN plants were cultivated together in the same environment. Therefore, the observed variation was likely attributed primarily to transgene movement into wild *B. juncea*. Among these proteins, storage protein was the most abundant (Fig. 3C), which support that storage protein is most sensitive to transgene insertion as discussed above. Proteins involved in cell defense and rescue also accounted for a large difference (Fig. 3C), in which three spots (70, 34 and 38) were identified as late embryogenesis abundant (LEA) proteins (Table 2). LEA proteins are thought to play an essential role in desiccation tolerance and vigor^43^,^44^,^45^, by replacing water, sequestering ions, removing ROS and/or stabilizing protein and membrane structure^45^,^46^. In this category, one protein, BnaC03g55840D, which is homologous to the 17.4 kDa class I heat shock protein (HSP), was newly accumulated in BJtBN seeds (Table 2). HSPs are well known to function as chaperones, which stabilize newly synthesized proteins to ensure correct folding or helping refold damaged proteins from stress.

It appears that there were few unintended effects of the GM event relative to natural hybridization effects, and data fell within the range of natural variability of hybridization. The number of protein spots (15) varied in abundance between BJtBN and BJBN seeds was far less than those (34) found between BJBN and BJ seeds. Among these 15 protein spots in BJtBN/BJBN, seven spots exhibited a same accumulation pattern between BJBN and BJ seeds (Fig. 5), and five spots (40, 32, 58, 72 and 38) were homologous to some of the differentially accumulated protein spots in BJBN/BJ (Tables 2 and 3). For example, spot 40 accumulated in higher abundance in BJtBN than in BJBN seeds is homologous to spots 41 and 42 whose abundance were higher in BJBN than in BJ seeds (Tables 2 and 3). Spots 32, 58 and 72, identified to be cruciferin storage protein accumulated in higher abundance in BJtBN seeds than BJBN seeds (Table 2). Similar accumulation pattern of cruciferin storage protein (spots 43, 46, 65, 66 and 73) could be observed between BJBN and BJ seeds (Table 3). Spot 38 (LEA protein family protein) in BJtBN/BJBN is homologous to spot 54 in BJBN/BJ, and they showed a same accumulation pattern in these two pair-wise comparisons (Tables 2 and 3). Herman and Price (2013) reviewed 20 years of research on unintended compositional changes in GM crops and found that the changes were small compared with those produced from traditional breeding and environmental factors^47^. Our results agree with this conclusion and extend it to the next generation of GM crops, namely hybrids with wild relatives.

Some of the differentially accumulated proteins identified by tBN/BN have similar functions as those identified by BJtBN/BJBN. For instances, some cruciferin storage proteins were differentially accumulated between BJtBN and BJBN seeds and between tBN and BN seeds (Tables 1 and 2). BnaC04g48420D (spot 5, Table 1) and LEA protein (spot 18, Table 1) in comparison of tBN *vs.* BN and At2g42560 (spots 34 and 38, Table 2) in comparison of BJtBN *vs.* BJBN had lower abundance in transgenic seeds than in non- transgenic seeds; these were members of the LEA protein family. Except for the functional similarity, however, none of the differentially accumulated protein spots were substantially identical between BJtBN/BJBN and tBN/BN seeds (Fig. 5). This indicates that the unintended effects resulting from insertion of exogenous gene in crops were not inherited in the next generation.

According to our experimental design and proteomic methods in this study, there were a few differentially accumulated proteins, but no clear patterns were detected between crops and between crop-wild relative generations. We concluded that potential unintended effects of this transgenic-wild system are negligible and within bounds of natural variation.

## Materials and Methods

### Plants

Seeds of wild brown mustard (*Brassica juncea*, 2*n* = 36, AABB) originating from a local field collection (Nanjing, China) were provided by Prof. S. Qiang, Nanjing Agricultural University. Transgenic oilseed rape (*B. napus* cv. Westar, 2*n* = 38, AACC) was produced by transforming with a pSAM12 plasmid containing genetically linked *gfp* (encoding a green fluorescent protein) and *Bt Cry1Ac* cassettes that are regulated by independent CaMV 35S promoters^48^.

Wild mustard, non-transgenic and transgenic *B. napus* were planted in three segregated greenhouses (natural light, average daily temperature varied between 20 and 30 °C) at the Chinese Research Academy of Environmental Sciences (Beijing, China) respectively. After emasculating the stamens of the maternal wild plants, one wild mustard plant (BJ) was hybridized with the pollen from one conventional *B. napus* (BN) to obtain F_1_ hybrid seeds (BJBN), and another wild mustard was crossed with the pollen of one transgenic *B. napus* (tBN) to form F_1_ hybrid seeds (BJtBN) (Fig. 1). The remainder of BN, tBN and BJ plants were self-pollinated (Fig. 1).

After maturation, seeds of BN, tBN, BJ, BJBN and BJtBN were harvested separately and dried in an air-conditioned room (28 °C, 45% humidity) until the water content reached a constant level (about 0.09 g H_2_O g^−1^ dry weight). The accumulation of Cry1Ac proteins in BN, tBN, BJ, BJBN and BJtBN seeds were detected using enzyme-linked immunosorbent assays (ELISA) from Agidia (EnviroLogix, USA), and the ELISA was performed following the manufacturer’s instructions. The *Cry1Ac* protein accumulated both in tBN and BJtBN (Supplemental Fig. S1). The negative controls (BN, BJ and BJBN) had no measurable *Bt* protein.

### Preparation of protein samples

Fifty seeds each of BN, tBN, BJ and their hybrids BJBN and BJtBN were homogenized in a total of 1.5 ml precooled extraction buffer containing 50 mM Tris-HCl (pH 7.5), 30% (w/v) sucrose, 10 mM EGTA, 1 mM PMSF, 1 mM DTT, and 1% (v/v) Triton X-100. The homogenate was centrifuged at 16 000 g for 10 min followed by at 32 000 g for 20 min at 4 °C. The resulting supernatant was mixed with two-fold volume of ice-cold Tris-HCl (0.1 M, pH 7.5) saturated phenol and shaken on ice for 30 min. After centrifugation at 16 000 g for 20 min, the phenol phase was collected and mixed with five times volume of precooled methanol saturated with (NH_4_)_2_SO_4._ Samples were incubated at –20 °C for over 6 hours and then centrifuged at 16 000 g for 5 min. The resulting pellets were rinsed four times with ice-cold acetone (100%) containing 13 mM DTT, and then lyophilized. Lyophilized protein samples were solubilized in lysis buffer composed of 7 M urea, 2 M thiourea, 2% (w/v) CHAPS, 20 mM DTT, and 0.5% (v/v) immobilized pH gradient (IPG) buffer (pH 3–10) and then used for determination of protein concentration by Bradford method^49^ using bovine serum albumin as the standard.

### 2-DE

Isoelectrofocusing (IEF) was performed using a PROTEAN i12 IEF system (Bio-Rad; Hercules, Piscataway, NJ, USA) and 17 cm Immobiline Dry Strips with a nonlinear pH gradient of 3–10 (Bio-Rad; Hercules, USA). The 400 μg protein sample dissolved in 300 μl lysis buffer was loaded onto the gel strip by passive rehydration: the gel strip was incubated at 20 °C for 16 h and then used for IEF. IEF was performed by applying a voltage of 250 V for 1 h, ramping to 500 V over 1 h, 2 000 V for 2 h, 10 000 V for 4 h and held at 10 000 V until a total of 60 kVh was reached. After IEF, the gel strip was reduced for 15 min with 65 mM DTT dissolved in 3 ml of equilibration buffer (6 M urea, 30% (v/v) glycerol, 2% (w/v) SDS, 50 mM Tris-HCl (pH 8.8) and 0.01% (w/v) bromophenol blue) and then alkylated with 2.5% (w/v) iodoacetamide in the same buffer for 15 min. The reduced and alkylated strip was placed onto a vertical SDS-polyacrylamide gel (12% resolving and 5% stacking), and the low-molecular-range marker (Bio-Rad) was loaded at one end of the strip. The strip was sealed with 0.5% (w/v) low-melting agarose in SDS buffer containing bromophenol blue before electrophoresis. Electrophoresis was performed at 15 °C in SDS electrophoresis buffer (pH 8.3) composed of 25 mM Tris base, 192 mM glycine and 1% (w/v) SDS, for 30 min at 25 mA and for 4.5 h at 40 mA. The gel was stained over 3 hours with 0.25% (w/v) CBB R-250 in 5:1:4 (v/v) methanol: acetic acid: water and destained in 2:1:7 (v/v) methanol: acetic acid: water solution with 3–5 changes of the solution, until a colorless background was achieved.

### Image analysis, in-gel digestion with trypsin and protein identification by MALDI-TOF-TOF mass spectrometry

The 2-D gels were scanned at a 300 dpi resolution with a UMAX Power Look 2100XL scanner (Maxium Tech., Taipei, China). Spot detection and gel comparisons were made with ImageMaster 2D Platimum, version 5.01 (GE Healthcare Bio-Science, Little Chalfont, UK). After automatic detection and matching, manual editing was carried out to correct the mismatched and unmatched spots.

Well-separated gels of the three independent biological replicates were used for proteomic comparisons. Spots were considered reproducible if they were detected in all the biological replicates. The normalized volume (based on the total spot volumes) of each spot was assumed to represent its accumulation abundance. Protein spot was considered to be differentially accumulated when the change was more than 1.5-fold and significant (p < 0.05, Students’ t-test). Protein spots, which varied in abundance were excised from the stained gels and digested according to Shevchenko *et al.*^50^ with minor modifications. Gel spots were washed and destained in 100 μl water followed by 100 μl 50% acetonitrile (ACN) (Fisher Scientific; Fair Lawn, NJ, USA) twice, respectively and then dehydrated with 50 μl 100% ACN at room temperature (RT). Dehydrated gel spots were first incubated in 10 mM DTT/100 mM NH_4_HCO_3_ for 45 min at 56 °C, followed by incubation with 55 mM iodoacetamide/100 mM NH_4_HCO_3_ for 30 min at RT in darkness. After a series of washes (as described above), the gel pieces were dehydrated in 100% ACN. Samples were subsequently rehydrated in digestion buffer containing 10 ng trypsin in 50 mM NH_4_HCO_3_ for 45 min at 4 °C. After removal of the excess of digestion buffer, the gel pieces were incubated in 50 mM NH_4_HCO_3_ for overnight at 37 °C.

Peptide mixtures were desalted on in-house made microcolumns packed with reverse phase material POROS 20R2 (Applied Biosystems, US) before MS analysis. The desalted samples were eluted from the column with 3 μl matrix solution (5 μg/μl α-cyano-4-hydroxycinnamic acid in 70% ACN, 0.1% TFA) directly onto the MALDI target plate (Opti-TOF 384 Well Insert, Applied Biosystems). MS and MS/MS spectra were acquired on an UltrafleXtreme MALDI time-of-flight/time-of-flight mass spectrometer (MALDI-TOF/TOF MS) (Bruker Daltonics, Germany) by use of FlexAnalysis 3.3 software. MS spectra in the range 700–3500 m/z were acquired automatically with external calibration to a standard β-lactoglobulin tryptic digest. Collision-induced dissociation was used for fragmentation of the 10 most intense precursor ions and was performed automatically with default calibration.

Protein identification was performed using Biotools 3.2 (Bruker Daltonics, Billerica, MA, US) by searching NCBInr database with Mascot 2.2.04 (Matrix Science). The following search parameters were used: Database -NCBInr (NCBInr 20140927), Taxonomy-Green plants (49886901 sequences; 17905752166 residues), maximum one missed cleavage, cysteine carbamidomethylation as a fixed modification, methionine oxidation and N-terminal acetylation as variable modifications, mass tolerance of 100 ppm in MS mode and 0.6 Da for MS/MS. Protein scores greater than 76 (in NCBInr database) were significant (p < 0.05). The identified protein name, accession number, Mascot score, sequence coverage, number of sequenced peptides (the peptides matched by the MS/MS spectra (ion score > 20) and of matched peptides (the peptides matched by the peptide mass fingerprinting (PMF) and theoretical protein mass and pI found in the databases are shown in Tables 1, 2, 3.

## Additional Information

**How to cite this article**: Liu, Y. *et al.* A proteomic analysis of seeds from *Bt*-transgenic *Brassica napus* and hybrids with wild *B. juncea. Sci. Rep.* **5**, 15480; doi: 10.1038/srep15480 (2015).

## Supplementary Material

Supplementary Information

## Figures and Tables

**Table 1:** Identified protein spots that are differentially accumulated between transgenic (tBN) and non-transgenic (BN) *Brassica napus* seeds.

**Spot ID**	**Accumulation Pattern**	**Ratio tBN/BN**	**Identified protein name**	**Accession number**	**Mascot score**	**Sequence coverage (%)**	**No. of sequenced/matched peptides**	**Exp. protein mass (kDa/pI)**	**Theor. protein mass (kDa/pI)**	**Function category**	**Function description**
24	H	2.8	BnaA05g33200D, partial (glyceraldehyde-3-phosphate dehydrogenase)	CDY49358	196	24	2/5	39/6.0	37/6.4	energy	glycolysis
25	H	2.5	BnaC07g48660D (cruciferin BnC1)	CDY65916	354	12	4/4	33/6.6	50.7/7.2	storage protein	storage protein
26	H	3.5	BnaC01g09900D (cruciferin subunit)	CDY38381	155	12	3/6	33/6.5	53/7.6	storage protein	storage protein
27	H	2.3	BnaA02g22500D (cruciferin)	CDY14908	564	19	6/8	32/8.2	51/8.2	storage protein	storage protein
29	H	3.2	PREDICTED: cruciferin CRU4	XP_009119353	212	22	3/7	30/8.2	52/6.8	storage protein	storage protein
30	H	4.7	cruciferin cru2/3 subunit	CAA40979	518	57	4/5	22/6.5	54/6.8	storage protein	storage protein
31	H	7.4	BnaA02g22500D (Cruciferin)	CDY14908	514	33	4/8	22/7.2	51/8.2	storage protein	storage protein
23	H	7.5	hypothetical protein MIMGU_mgv1a005250 mg (catalase isozyme 3 )	EYU30275	249	8	2/3	56/6.9	57/6.9	cell defense & rescue	detoxification
2	L	0.4	PREDICTED: lysosomal alpha-mannosidase	XP_009131373	1410	26	14/25	75/6.3	116/6.4	metabolism	sugar & polysaccharide
12	L	D	BnaC06g06810D (Myrosinase-associated protein)	CDY36628	846	32	8/9	42/8.3	43/8.1	metabolism	lipid
14	L	0.2	myrosinase-associated protein	AAC49181	680	36	7/9	28/6.1	42/8.5	metabolism	lipid
7	L	0.5	BnaA04g15750D (strictosidine synthase 1-like)	CDY30558	206	39	2/9	58/7.3	42/6.7	metabolism	others
3	L	0.2	BnaC05g18490D (phosphoglucomutase)	CDY04740	100	4	1/2	72/6.1	64/5.9	energy	glycolysis
6	L	0.4	glyceraldehyde-3-phosphate dehydrogenase 2	ACS68203	318	37	4/8	61/7.3	37/7.7	energy	glycolysis
4	L	0.5	BnaA07g00860D (malic enzyme)	CDY35901	99	10	1/2	57/6.3	65/6.5	energy	pentose-phosphate shunt
8	L	D	Cruciferin CRU4	P33522	606	25	9/10	57/7.7	51/7.7	storage protein	storage protein
20	L	0.2	BnaC03g61870D (provicilin)	CDY35473	592	16	5/8	24/5.3	65/5.4	storage protein	storage protein
15	L	D	BnaC03g41580D (probable inactive serine/threonine-protein kinase fnkC isoform X2)	CDY36040	793	26	6/13	38/8.4	44/7.7	signal transduction	kinases
1	L	0.3	PREDICTED: embryonic protein DC-8	XP_009142073	797	15	7/8	80/6.1	73/6.0	cell structure	others
10	L	0.6	PREDICTED: epithiospecifier protein-like	XP_009107061	180	48	2/12	44/5.5	38/5.5	cell defense & rescue	defense-related
11	L	0.2	BnaC06g10450D (epithiospecifier protein-like)	CDY30681	442	22	3/6	44/5.4	39/5.3	cell defense & rescue	defense-related
5	L	0.3	BnaC04g48420D (seed biotin-containing protein SBP65-like isoform X1)	CDY14845	174	7	2/4	68/7.8	68/5.9	cell defense & rescue	stress response
18	L	0.5	late embryogenesis-abundant protein	BAB88878	146	27	1/5	29/8.2	25/9.0	cell defense & rescue	stress response
16	L	D	BnaA08g25110D (glyoxalase I)	CDY21014	297	28	5/9	34/5.5	32/5.3	cell defense & rescue	detoxification

Spot ID is the spot number shown in Fig. 2; accumulation pattern indicates the spot whose abundance is higher (H) or lower (L) in tBN seeds than BN seeds; Ratio tBN/BN, normalized spot volume in tBN seeds divided by the normalized volume in BN seeds; No. of sequence peptides, the peptides matched by the MS/MS spectra (Ion score > 20). No. of matched peptides, the peptides matched by the PMF; exp. protein mass, experimental protein mass; theor. protein mass, theoretical protein mass. D: disappeared. Proteins in the bracket are the homologous proteins of the identified proteins.

**Table 2:** Identified protein spots that are differentially accumulated between hybrids of *B. juncea* (BJ) with transgenic *B. napus* (BJtBN) and of BJ with non-transgenic *B. napus* (BJBN).

**Spot ID**	**Accumulation Pattern**	**Ratio BJtBN/BJBN**	**Identified protein name**	**Accession number**	**Mascot score**	**Sequence coverage (%)**	**No. of sequenced/matched peptides**	**Exp. protein mass (kDa/pI)**	**Theor. protein mass (kDa/pI)**	**Function category**	**Function description**
40	H	3.4	myrosinase, thioglucoside glucohydrolase	CAA11412	1380	32	14/20	63/5.9	63/6.1	metabolism	sugar & polysaccharide
41	H	4.5	myrosinase 2	ADP24127	719	36	5/17	63/6.0	63/6.5	metabolism	sugar & polysaccharide
32	H	4.1	BnaC01g09900D (cruciferin subunit)	CDY38381	581	17	7/9	93/7.8	53/7.6	storage protein	storage protein
43	H	1.9	Cruciferin CRU4	P33522	519	28	7/10	55/7.6	52/7.7	storage protein	storage protein
58	H	2.0	PREDICTED: 12S seed storage protein CRD	XP_009118685	821	31	7/10	31/6.1	49/5.7	storage protein	storage protein
72	H	A	cruciferin storage protein	BAJ78781	126	18	2/2	17/5.6	11/8.8	storage protein	storage protein
73	H	2.2	cruciferin storage protein	BAJ78781	195	25	2/2	17/6.1	11/8.8	storage protein	storage protein
67	H	A	BnaC03g55840D (17.4 kDa class I heat shock protein)	CDX86069	261	31	2/3	23/5.8	18/5.8	cell defense & rescue	stress response
70	H	1.9	late embryogenesis-abundant protein	ABB55259	265	29	3/4	18/6.0	10/5.9	cell defense & rescue	stress response
39	L	0.4	PREDICTED: beta-glucosidase 19 isoform X1	XP_009109612	1060	38	10/16	69/6.7	61/6.4	metabolism	sugar & polysaccharide
53	L	0.6	PREDICTED: provicilin	XP_009108832	145	3	1/1	38/6.6	55/6.6	storage protein	storage protein
34	L	0.4	At2g42560 (late embryogenesis abundant domain-containing protein)	XP_002881860	119	4	1/2	90/6.6	67/6.0	cell defense & rescue	stress response
38	L	0.1	At2g42560 (late embryogenesis abundant domain-containing protein)	XP_002881860	130	11	1/5	89/6.7	67/6.0	cell defense & rescue	stress response
61	L	0.4	BnaA05g15190D (stress responsive A/B barrel domain protein)	CDY43909	364	33	3/5	28/4.9	23/4.9	cell defense & rescue	stress response
59	L	0.2	PREDICTED: uncharacterized protein LOC103844119	XP_009119147	144	18	3/3	30/6.1	27/5.8	unknown	unknown

Spot ID is the spot number shown in Fig. 3; accumulation pattern indicates the spot whose abundance is higher (H) or lower (L) in BJtBN seeds than BJBN seeds; Ratio BJtBN/BJBN, normalized spot volume in BJtBN seeds divided by the normalized volume in BJBN seeds; No. of sequence peptides/matched peptides, exp. protein mass and theo. protein mass: theoretical protein mass are described in Table 1. A: appeared. Proteins in the bracket are the homologous proteins of the identified proteins.

**Table 3:** Identified protein spots that are differentially accumulated between *B. juncea* (BJ) and hybrids of BJ with non-transgenic *B. napus* (BJBN).

**Spot ID**	**Accumulation Pattern**	**Ratio BJBN/BJ**	**Identified protein name**	**Accession number**	**Mascot score**	**Sequence coverage (%)**	**No. of sequenced/matched peptides**	**Exp. protein mass (kDa/pI)**	**Theor. protein mass (kDa/pI)**	**Function category**	**Function description**
35	H	7.9	cobalamin-independent methionine synthase	XP_002871787	1170	38	11/22	83/6.2	85/6.1	metabolism	amino acid
37	H	8.1	hypothetical protein CARUB_v10000282 mg (cobalamin-independent methionine synthase)	XP_006286451	1380	39	13/20	84/6.3	85/6.2	metabolism	amino acid
39	H	3.1	PREDICTED: beta-glucosidase 19 isoform X1	XP_009109612	1060	38	10/16	69/6.7	61/6.4	metabolism	sugar & polysaccharide
41	H	1.8	myrosinase 2	ADP24127	719	36	5/17	63/6.0	63/6.5	metabolism	sugar & polysaccharide
42	H	2.5	myrosinase	ABQ42337	1460	29	12/14	63/6.1	61/6.3	metabolism	sugar & polysaccharide
71	H	7.5	BnaC03g70690D (nucleoside diphosphate kinase 1)	CDY10545	234	56	3/7	18/6.2	17/6.3	metabolism	nucleotide
51	H	A	BnaCnng26110D (strictosidine synthase 1-like)	CDY54042	1060	52	9/16	48/6.4	42/6.1	metabolism	others
45	H	2.4	ATPase alpha subunit	ABO86590	1080	29	9/14	57/6.2	55/6.0	energy	respiration
33	H	2.1	PREDICTED: elongation factor 2-like	XP_009129918	1230	30	13/18	99/6.3	95/5.9	protein synthesis	translational factor
43	H	1.7	Cruciferin CRU4	P33522	519	28	7/10	55/7.6	52/7.7	storage protein	storage protein
46	H	A	BnaC07g48660D (cruciferin BnC1)	CDY65916	590	33	6/10	55/6.7	51/7.2	storage protein	storage protein
53	H	A	PREDICTED: provicilin	XP_009108832	145	3	1/1	38/6.6	55/6.6	storage protein	storage protein
65	H	6.0	cruciferin cru2/3 subunit	CAA40979	300	73	3/5	22/6.0	54/6.8	storage protein	storage protein
66	H	4.4	cruciferin cru2/3 subunit	CAA40979	617	57	4/5	22/6.6	54/6.8	storage protein	storage protein
68	H	1.5	hypothetical protein CARUB_v10012738 mg (12S seed storage protein )	XP_006279075	437	12	5/5	22/6.1	52/6.7	storage protein	storage protein
73	H	2.2	cruciferin storage protein	BAJ78781	195	25	2/2	17/6.1	11/8.8	storage protein	storage protein
36	H	4.2	PREDICTED: embryonic protein DC-8	XP_009142073	727	16	6/8	84/6.1	73/6.0	cell structure	others
44	H	2.6	PREDICTED: myrosinase-binding protein-like At3g16440	XP_009109600	923	47	8/24	57/6.2	50/6.1	cell defense & rescue	defense-related
55	H	A	BnaC05g11200D (myrosinase-binding protein-like)	CDY53520	920	49	8/14	35/6.0	31/5.9	cell defense & rescue	defense-related
38	H	12.6	At2g42560 (late embryogenesis abundant domain-containing protein)	XP_002881860	130	11	1/5	89/6.7	67/6.0	cell defense & rescue	stress response
49	H	A	PREDICTED: cytosolic isocitrate dehydrogenase [NADP]	XP_009127460	1480	73	14/32	52/6.4	46/6.3	cell defense & rescue	stress response
70	H	2.0	late embryogenesis-abundant protein	ABB55259	265	29	3/4	18/6.0	10/5.9	cell defense & rescue	stress response
48	H	2.6	PREDICTED: alcohol dehydrogenase class-3	XP_009101752	996	45	9/10	52/6.7	42/6.5	cell defense & rescue	detoxification
57	H	1.7	peroxiredoxin antioxidant	AAF61460	690	74	6/9	31/6.2	24/6.0	cell defense & rescue	detoxification
47	L	0.2	PREDICTED: cruciferin CRU4	XP_009119353	380	19	5/8	53/6.8	52/6.8	storage protein	storage protein
62	L	0.5	cupin domain-containing protein	NP_180416	248	9	3/4	25/5.1	56/5.8	storage protein	storage protein
64	L	0.3	BnaA07g13950D (vicilin-like antimicrobial peptides 2-2)	CDY04569	222	11	2/3	23/6.0	52/6.0	cell defense & rescue	defense-related
34	L	0.3	At2g42560 (late embryogenesis abundant domain-containing protein)	XP_002881860	119	4	1/2	90/6.6	67/6.0	cell defense & rescue	stress response
54	L	0.3	PREDICTED: late embryogenesis abundant protein D-34-like	XP_009145325	140	42	1/6	36/4.7	27/4.7	cell defense & rescue	stress response
61	L	0.5	BnaA05g15190D (stress responsive A/B barrel domain protein)	CDY43909	364	33	3/5	28/4.9	23/4.9	cell defense & rescue	stress response
69	L	0.4	BnaC09g44630D (17.6 kDa class II heat shock protein)	CDX97162	288	34	2/3	21/6.2	17/6.2	cell defense & rescue	stress response
50	L	0.5	PREDICTED: alcohol dehydrogenase class-3	XP_009101752	854	45	9/12	52/6.6	42/6.5	cell defense & rescue	detoxification
60	L	0.2	Fe superoxide dismutase 1, partial	ADR01109	194	12	2/3	42/8.1	22/5.8	cell defense & rescue	detoxification
59	L	0.4	PREDICTED: uncharacterized protein LOC103844119	XP_009119147	144	18	3/3	30/6.1	27/5.8	unknown	unknown

Spot ID is the spot number shown in Fig. 4; accumulation pattern indicates the spot whose abundance is higher (H) or lower (L) in BJBN seeds than BJ seeds; Ratio BJBN/BJ, normalized spot volume in BJBN seeds divided by the normalized volume in BJ seeds; No. of sequence peptides/matched peptides, exp. protein mass and theo. protein mass: theoretical protein mass are described in Table 1. A: appeared. Proteins in the bracket are the homologous proteins of the identified proteins.

## References

[b1] Cellini F. *et al.* Unintended effects and their detection in genetically modified crops. Food Chem Toxicol 42, 1089–1125 (2004). 15123383 10.1016/j.fct.2004.02.003

[b2] Garcia-Canas V., Simo C., Leon C., Ibanez E. & Cifuentes A. MS-based analytical methodologies to characterize genetically modified crops. Mass spectrometry reviews 30, 396–416 (2011). 21500243 10.1002/mas.20286

[b3] Gong C. Y. & Wang T. Proteomic evaluation of genetically modified crops: Current status and challenges. Front Plant Sci 4, 1–8 (2013). 23471542 10.3389/fpls.2013.00041 PMC3590489

[b4] Kuiper H. A., Kleter G. A., Noteborn H. P. J. M. & Kok E. J. Assessment of the food safety issues related to genetically modified foods. Plant J 27, 503–528 (2001). 11576435 10.1046/j.1365-313x.2001.01119.x

[b5] Ricroch A. E., Berge J. B. & Kuntz M. Evaluation of genetically engineered crops using transcriptomic, proteomic, and metabolomic profiling techniques. Plant Physiol 155, 1752–1761 (2011). 21350035 10.1104/pp.111.173609 PMC3091128

[b6] Di Carli M. *et al.* Leaf proteome analysis of transgenic plants expressing antiviral antibodies. J Proteome Res 8, 838–848 (2009). 19099506 10.1021/pr800359d

[b7] OECD. Safety Evaluation of Foods Derived by Modern Biotechnology: Concepts and Principles. Paris: Organisation for Economic Co-operation and Development (1993).

[b8] Barros E. *et al.* Comparison of two GM maize varieties with a near-isogenic non-GM variety using transcriptomics, proteomics and metabolomics. Plant Biotechnol J 8, 436–451 (2010). 20132517 10.1111/j.1467-7652.2009.00487.x

[b9] Coll A., Nadal A., Rossignol M., Puigdomenech P. & Pla M. Proteomic analysis of MON810 and comparable non-GM maize varieties grown in agricultural fields. Transgenic Res 20, 939–949 (2011). 20972621 10.1007/s11248-010-9453-y

[b10] Corpillo D. *et al.* Proteomics as a tool to improve investigation of substantial equivalence in genetically modified organisms: The case of a virus-resistant tomato. Proteomics 4, 193–200 (2004). 14730681 10.1002/pmic.200300540

[b11] Gong C. Y., Li Q., Yu H. T., Wang Z. Z. & Wang T. Proteomics insight into the biological safety of transgenic modification of rice as compared with conventional genetic breeding and spontaneous genotypic variation. J Proteome Res 11, 3019–3029 (2012). 22509807 10.1021/pr300148w

[b12] Kim J. K. *et al.* Unintended polar metabolite profiling of carotenoid-biofortified transgenic rice reveals substantial equivalence to its non-transgenic counterpart. Plant Biotechnol Rep 7, 121–128 (2013).

[b13] Kogel K. H. *et al.* Transcriptome and metabolome profiling of field-grown transgenic barley lack induced differences but show cultivar-specific variances. Proc Natl Acad Sci USA 107, 6198–6203 (2010). 20308540 10.1073/pnas.1001945107 PMC2851944

[b14] Zhao X. X. *et al.* Unintended changes in genetically modified rice expressing the lysine-rich fusion protein gene revealed by a proteomics approach. J Integr Agr 12, 2013–2021 (2013).

[b15] Albo A. G. *et al.* Proteomic analysis of a genetically modified maize flour carrying *Cry1Ab* gene and comparison to the corresponding wild-type. Maydica 52, 443–455 (2007).

[b16] Zolla L., Rinalducci S., Antonioli P. & Righetti P. G. Proteomics as a complementary tool for identifying unintended side effects occurring in transgenic maize seeds as a result of genetic modifications. J Proteome Res 7, 1850–1861 (2008). 18393457 10.1021/pr0705082

[b17] Sestili F. *et al.* Comparative proteomic analysis of kernel proteins of two high amylose transgenic durum wheat lines obtained by biolistic and Agrobacterium-mediated transformations. J Cereal Sci 58, 15–22 (2013).

[b18] Chen H. C. *et al.* Unintended changes in protein expression revealed by proteomic analysis of seeds from transgenic pea expressing a bean alpha-amylase inhibitor gene. Proteomics 9, 4406–4415 (2009). 19725077 10.1002/pmic.200900111

[b19] Islam N., Campbell P. M., Higgins T. J. V., Hirano H. & Akhurst R. J. Transgenic peas expressing an alpha-amylase inhibitor gene from beans show altered expression and modification of endogenous proteins. Electrophoresis 30, 1863–1868 (2009). 19517428 10.1002/elps.200800717

[b20] Rocco M. *et al.* The expression of tomato prosystemin gene in tobacco plants highly affects host proteomic repertoire. J Proteomics 71, 176–185 (2008). 18617145 10.1016/j.jprot.2008.04.003

[b21] Cao D. *et al.* Stable *Bacillus thuringiensis* transgene introgression from *Brassica napus* to wild mustard *B. juncea*. Plant Sci 227, 45–50 (2014). 25219305 10.1016/j.plantsci.2014.06.018

[b22] Ellstrand N. C. *et al.* Introgression of crop alleles into wild or weedy populations. Annu Rev Ecol Evol S 44, 325–345 (2013).

[b23] Gueritaine G., Sester M., Eber F., Chevre A. M. & Darmency H. Fitness of backcross six of hybrids between transgenic oilseed rape (*Brassica napus*) and wild radish (*Raphanus raphanistrum*). Mol Ecol 11, 1419–1426 (2002). 12144662 10.1046/j.1365-294x.2002.01540.x

[b24] Snow A. A. *et al.* Long-term persistence of crop alleles in weedy populations of wild radish (*Raphanus raphanistrum*). New Phytol 186, 537–548 (2010). 20122132 10.1111/j.1469-8137.2009.03172.x

[b25] Stewart C. N., Halfhill M. D. & Warwick S. I. Transgene introgression from genetically modified crops to their wild relatives. Nat Rev Genet 4, 806–817 (2003). 14526376 10.1038/nrg1179

[b26] Liu Y. B. *et al.* Consequences of gene flow between oilseed rape (*Brassica napus*) and its relatives. Plant Sci 211, 42–51 (2013). 23987810 10.1016/j.plantsci.2013.07.002

[b27] Darmency H. The impact of hybrids between genetically-modified crop plants and their related species - introgression and weediness. Mol Ecol 3, 37–40 (1994).

[b28] Hilbeck A. Implications of transgenic, insecticidal plants for insect and plant biodiversity. Perspect Plant Ecol 4, 43–61 (2001).

[b29] Laughlin K. D., Power A. G., Snow A. A. & Spencer L. J. Risk assessment of genetically engineered crops: Fitness effects of virus-resistance transgenes in wild *Cucurbita pepo*. Ecol Appl 19, 1091–1101 (2009). 19688918 10.1890/08-0105.1

[b30] Liu Y. B., Wei W., Ma K. P. & Darmency H. Backcrosses to *Brassica napus* of hybrids between *B. juncea* and *B. napus* as a source of herbicide-resistant volunteer-like feral populations. Plant Sci 179, 459–465 (2010). 21802604 10.1016/j.plantsci.2010.07.005

[b31] Vacher C., Kossler T. M., Hochberg M. E. & Weis A. E. Impact of interspecific hybridization between crops and weedy relatives on the evolution of flowering time in weedy phenotypes. Plos One 6 (2011). 10.1371/journal.pone.0014649 PMC3033403 21304909

[b32] Vacher C. *et al.* Impact of ecological factors on the initial invasion of *Bt* transgenes into wild populations of birdseed rape (*Brassica rapa*). Theor Appl Genet 109, 806–814 (2004). 15340690 10.1007/s00122-004-1696-7

[b33] Bevan M. *et al.* Analysis of 1.9 Mb of contiguous sequence from chromosome 4 of *Arabidopsis thaliana*. Nature 391, 485–488 (1998). 9461215 10.1038/35140

[b34] Bewley J. D., Bradford K. J., Hilborst H. W. M. & Nonogaki H. Seeds-Physiology of Development, Germination and Dormancy. (Springer, 2013).

[b35] Tan-Wilson A. L. & Wilson K. A. Mobilization of seed protein reserves. Physiol Plantarum 145, 140–153 (2012). 10.1111/j.1399-3054.2011.01535.x 22017287

[b36] Wang W. Q., Liu S. J., Song S. Q. & Møller I. M. Proteomics of seed development, desiccation tolerance, germination and vigor. Plant Physiol Biochem 86, 1–15 (2015). 25461695 10.1016/j.plaphy.2014.11.003

[b37] Bailly C. Active oxygen species and antioxidants in seed biology. Seed Sci Res 14, 93–107 (2004).

[b38] Møller I. M. Plant mitochondria and oxidative stress: Electron transport, NADPH turnover, and metabolism of reactive oxygen species. Annu Rev Plant Phys 52, 561–591 (2001). 10.1146/annurev.arplant.52.1.561 11337409

[b39] Miernyk J. A. & Hajduch M. Seed proteomics. J Proteomics 74, 389–400 (2011). 21172463 10.1016/j.jprot.2010.12.004

[b40] Brandao A. R., Barbosa H. S. & Arruda M. A. Z. Image analysis of two-dimensional gel electrophoresis for comparative proteomics of transgenic and non-transgenic soybean seeds. J Proteomics 73, 1433–1440 (2010). 20123049 10.1016/j.jprot.2010.01.009

[b41] Liu Y. B. *et al.* The effects of seed size on hybrids formed between oilseed rape (*Brassica Napus*) and wild brown mustard (*B. juncea*). Plos One 7, e39705 (2012). 22745814 10.1371/journal.pone.0039705 PMC3382164

[b42] Song X. L., Wang Z., Zuo J., Huangfu C. H. & Qiang S. Potential gene flow of two herbicide-tolerant transgenes from oilseed rape to wild *B. juncea* var. *gracilis*. Theor Appl Genet 120, 1501–1510 (2010). 20151105 10.1007/s00122-010-1271-3

[b43] Boudet J. *et al.* Comparative analysis of the heat stable proteome of radicles of *Medicago truncatula* seeds during germination identifies late embryogenesis abundant proteins associated with desiccation tolerance. Plant Physiol 140, 1418–1436 (2006). 16461389 10.1104/pp.105.074039 PMC1435805

[b44] Chatelain E. *et al.* Temporal profiling of the heat-stable proteome during late maturation of *Medicago truncatula* seeds identifies a restricted subset of late embryogenesis abundant proteins associated with longevity. Plant Cell Environ 35, 1440–1455 (2012). 22380487 10.1111/j.1365-3040.2012.02501.x

[b45] Cuming A. C. LEA proteins. (Kluwer Academic Press, 1999).

[b46] Battaglia M., Olvera-Carrillo Y., Garciarrubio A., Campos F. & Covarrubias A. A. The enigmatic LEA proteins and other hydrophilins. Plant Physiol 148, 6–24 (2008). 18772351 10.1104/pp.108.120725 PMC2528095

[b47] Herman R. A. Unintended compositional changes in genetically modified (GM) crops: 20 years of research. J Agr Food Chem 61, 11695–11701 (2013). 23414177 10.1021/jf400135r

[b48] Halfhill M. D., Richards H. A., Mabon S. A. & Stewart C. N. Expression of *GFP* and *Bt* transgenes in *Brassica napus* and hybridization with *Brassica rapa*. Theor Appl Genet 103, 659–667 (2001).

[b49] Bradford M. M. A rapid and sensitive method for the quantitation of microgram quantities of protein utilizing the principle of protein-dye binding. Anal Chem 72, 248–254 (1976). 10.1016/0003-2697(76)90527-3 942051

[b50] Shevchenko A., Wilm M., Vorm O. & Mann M. Mass spectrometric sequencing of proteins from silver stained polyacrylamide gels. Anal Chem 68, 850–858 (1996). 8779443 10.1021/ac950914h

