# Supplementary material for: A proteomic analysis of seeds from Bt-transgenic Brassica napus and hybrids with wild B. juncea
Source: Sci Rep. 2015 Oct 21;5:15480. doi: 10.1038/srep15480 (PMC4614387; doi:10.1038/srep15480)
Supplement: Supplementary Information [file srep15480-s1.doc]

A proteomic analysis of seeds from *Bt*-transgenic *Brassica napus* and hybrids with wild *B. juncea*

Yongbo Liu^1,†,*^, Ying-Xue Zhang^2,4,†^, Song-Quan Song^2^, Junsheng Li^1^, C. Neal Stewart, Jr^5^, Wei Wei^3^, Yujie Zhao^1^, Wei-Qing Wang^2,*^

^1^ State Key Laboratory of Environmental Criteria and Risk Assessment, Chinese Research Academy of Environmental Sciences, 8 Dayangfang, Beijing 100012, China

^2^Key Laboratory of Plant Resources and Beijing Botanical Garden, Institute of Botany, Chinese Academy of Sciences, Beijing 100093, China

^3^State Key Laboratory of Vegetation and Environmental Change, Institute of Botany, Chinese Academy of Sciences, Beijing 100093, China

^4^College of Chemistry and Chemical Engineering, Henan University, Kaifeng, 475001, China

^5^Department of Plant Sciences, University of Tennessee, 2431 Joe Johnson Drive, Knoxville, TN 37996-4561, USA

^†^Contribution equally

^*^Author for Correspondence:

Emails: [liuyb@craes.org.cn](mailto:liuyb@craes.org.cn) (Y.L.); [wwq0814@ibcas.ac.cn](mailto:wwq0814@ibcas.ac.cn) (W.W.).

Tel/Fax: +86 10 84910906 (Y.L.); +86 10 62836049(W.W.).

**Supplementary information**


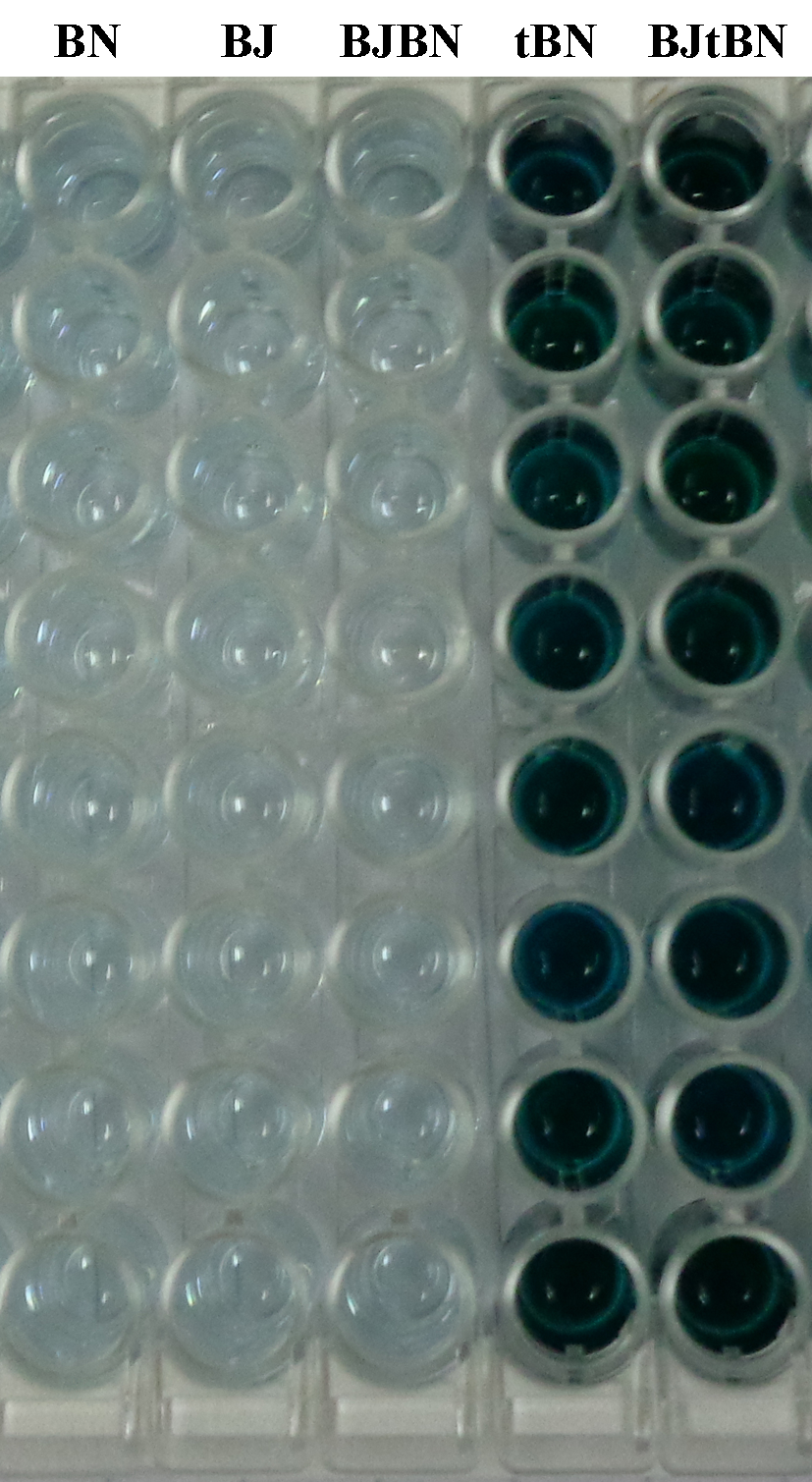


**Figure S1.** ELISA detection of Cry1Ac protein in transgenic and non-transgenic seeds. Columns from left to right were non-transgenic *Brassica napus* (BN), wild *B. juncea* (BJ), non-transgenic hybrids (BJBN) from BJ with BN, transgenic *B. napus* (tBN) and transgenic hybrids from BJ with tBN (BJtBN), respectively. Blue color indicates the expression of Cry1Ac proteins.

| **Table S1. Volume (%), ratio and p value of the differentially accumulated spots between non-transgenic (BN) and transgenic (tBN) oilseed rape seeds.** | | | | | | | | |
| --- | --- | --- | --- | --- | --- | --- | --- | --- |
| Spot ID | BN-1 | BN-2 | BN-3 | tBN-1 | tBN-2 | tBN-3 | tBN/BN | p value |
| 1 | 0.064087 | 0.0639162 | 0.0870187 | 0.0259007 | 0.0154595 | 0.0319814 | 0.3 | 0.006 |
| 2 | 0.0836432 | 0.0885253 | 0.0629565 | 0.0357088 | 0.030029 | 0.0224207 | 0.4 | 0.005 |
| 3 | 0.0355837 | 0.0450198 | 0.0567595 | 0.0135196 | 0.0148014 | 0 | 0.2 | 0.009 |
| 4 | 0.0424355 | 0.0563124 | 0.0423582 | 0.02534684 | 0.01564654 | 0.02546878 | 0.5 | 0.012 |
| 5 | 0.0906701 | 0.0600877 | 0.0746084 | 0.0258729 | 0.0146549 | 0.0308569 | 0.3 | 0.007 |
| 6 | 0.0627688 | 0.0504496 | 0.0585311 | 0.0137517 | 0.0231932 | 0.0235342 | 0.4 | 0.002 |
| 7 | 0.105941 | 0.0754502 | 0.0671927 | 0.0222746 | 0.0564797 | 0.0375307 | 0.5 | 0.046 |
| 8 | 0.0964329 | 0.0791905 | 0.0591458 | 0 | 0 | 0 | D* | 0.002 |
| 9 | 0.167124 | 0.160603 | 0.195393 | 0.0347614 | 0.0560732 | 0.0598321 | 0.3 | 0.001 |
| 10 | 0.2979 | 0.45167 | 0.374752 | 0.195664 | 0.209305 | 0.254867 | 0.6 | 0.032 |
| 11 | 0.178573 | 0.176968 | 0.141437 | 0.0294873 | 0.0322393 | 0.0538733 | 0.2 | 0.001 |
| 12 | 0.236801 | 0.150022 | 0.223911 | 0 | 0 | 0 | D | 0.002 |
| 13 | 0.0248659 | 0.0531209 | 0.0519464 | 0 | 0 | 0 | D | 0.009 |
| 14 | 0.116805 | 0.195299 | 0.115246 | 0.0526828 | 0.0247805 | 0 | 0.2 | 0.019 |
| 15 | 0.116357 | 0.0863961 | 0.0856765 | 0 | 0 | 0 | D | 0.001 |
| 16 | 0.140132 | 0.177784 | 0.0895237 | 0 | 0 | 0 | D | 0.006 |
| 17 | 0.19952 | 0.135129 | 0.153155 | 0.0728083 | 0.115235 | 0.0646005 | 0.5 | 0.034 |
| 18 | 0.204298 | 0.217685 | 0.18371 | 0.0709214 | 0.105788 | 0.112553 | 0.5 | 0.003 |
| 19 | 0.151094 | 0.15799 | 0.124698 | 0.0359137 | 0.0674364 | 0.0787427 | 0.4 | 0.007 |
| 20 | 0.171836 | 0.0900931 | 0.0896162 | 0.0223465 | 0.034646 | 0.024615169 | 0.2 | 0.031 |
| 21 | 0.362395 | 0.240307 | 0.265097 | 0 | 0 | 0 | D | 0.001 |
| 22 | 0.461956 | 0.427119 | 0.338683 | 0.150085 | 0.14643 | 0.166221 | 0.4 | 0.002 |
| 23 | 0.00904601 | 0.0154207 | 0.00973003 | 0.0824518 | 0.123626 | 0.0517684 | 7.5 | 0.023 |
| 24 | 0.0595898 | 0.0204264 | 0.037143 | 0.105382 | 0.094233 | 0.13226 | 2.8 | 0.011 |
| 25 | 0.286219 | 0.419244 | 0.470746 | 0.807506 | 0.898237 | 1.1813 | 2.5 | 0.010 |
| 26 | 0.080471 | 0.128222 | 0.239678 | 0.515397 | 0.465398 | 0.566161 | 3.5 | 0.003 |
| 27 | 0.126481 | 0.203846 | 0.108119 | 0.315193 | 0.400203 | 0.279948 | 2.3 | 0.016 |
| 28 | 0.0600675 | 0.0494854 | 0.0590492 | 0.176845 | 0.198804 | 0.223464 | 3.6 | 0.000 |
| 29 | 0.0365467 | 0.0972045 | 0.0609718 | 0.145336 | 0.236108 | 0.238516 | 3.2 | 0.016 |
| 30 | 0.370583 | 0.526983 | 0.436771 | 1.65316 | 2.17268 | 2.42277 | 4.7 | 0.002 |
| 31 | 0.193467 | 0.249091 | 0.184713 | 1.06771 | 1.84018 | 1.74411 | 7.4 | 0.005 |
| *D, disappeared; spot ID, spot numbers shown in Fig. 2; BN-1-3, replicates 1 to 3 of non transgenic oilseed rape seeds; tBN-1-3, replicates 1 to 3 of transgenic oilseed rape seeds; tBN/BN, normalized spot volume in tBN seeds divided by the normalized volume in BN seeds. | | | | | | | | |

| **Table S2. Identified differentially accumulated protein spots matched to two proteins.** | | | | | | | | |
| --- | --- | --- | --- | --- | --- | --- | --- | --- |
| Functional groups | Spot ID | Identified protein name | Accession number | Mascot score | sequence coverage(%) | No. of sequenced/matched peptides | Exp. protein mass(kDa)/pI | Theor. protein mass(kDa)/pI |
| Stress response | 13 | BnaC03g28700D | CDY05072 | 170 | 11 | 2/3 | 43/5.5 | 39.800/5.40 |
| Lipid | 13 | PREDICTED: acyl-[acyl-carrier-protein] desaturase, chloroplastic-like isoform X1 | XP_006472246 | 99 | 18 | 2/5 | 43/5.5 | 45.302/6.11 |
| Lipid | 17 | aspartic proteinase | AAC49730 | 492 | 22 | 5/8 | 30/5.3 | 53.047/5.29 |
| Nucleotide | 17 | BnaC06g19660D | CDX79337 | 400 | 21 | 4/12 | 30/5.3 | 87.873/5.08 |
| Defense | 21 | BnaA07g13950D | CDY04569 | 149 | 10 | 2/4 | 23/5.7 | 52.314/6.00 |
| Storage proteins | 21 | BnaA08g15380D | CDY24772 | 120 | 13 | 2/5 | 23/5.7 | 62.489/5.53 |
| Unknown | 28 | PREDICTED: uncharacterized protein LOC103865173 | XP_009141204 | 438 | 47 | 4/10 | 31/5.7 | 27.179/5.69 |
| Protein synthesis | 28 | BnaC03g45100D | CDY50408 | 245 | 15 | 3/3. | 31/5.7 | 27.269/5.48 |
| Lipid | 56 | aspartic protease | AAB03108 | 270 | 21 | 3/7 | 32/5.1 | 54.944/5.12 |
| Nucleotide | 56 | PREDICTED: nudix hydrolase 3 | XP_009104506 | 246 | 8 | 3/5 | 32/5.1 | 87.678/5.20 |
| Spots 13, 17, 21 and 28 were found to accumulated differentially between non-transgenic (BN) and transgenic (tBN) *Brassica napus* seeds, while spot 56 were found to accumulated differentially among seeds of wild *B. juncea* (BJ), hybrids of BJ with BN (BJBN) and of BJ with tBN (BJtBN). Spot ID is the spot number shown in Figures 2-4; No. of sequence peptides, the peptides matched by the MS/MS spectra (Ion score > 20). No. of matched peptides, the peptides matched by the PMF; exp. protein mass, experimental protein mass; theor. protein mass, theoretical protein mass. | | | | | | | | |

| **Table S3. Volume (%), ratio and p value of the differentially accumulated protein spots between hybrids of wild mustard (BJ) with nontransgenic (BJBN) and BJ with transgenic (BJtBN) oilseed rape seeds.** | | | | | | | | |
| --- | --- | --- | --- | --- | --- | --- | --- | --- |
| Spot ID | BJBN-1 | BJBN-2 | BJBN-3 | BJtBN-1 | BJtBN-2 | BJtBN-3 | BJtBN/BJBN | p value |
| 32 | 0.016 | 0.025 | 0.024 | 0.073 | 0.118 | 0.070 | 4.1 | 0.014 |
| 34 | 0.106 | 0.098 | 0.088 | 0.034 | 0.042 | 0.027 | 0.4 | 0.001 |
| 38 | 0.114 | 0.135 | 0.091 | 0.017 | 0.013 | 0.019 | 0.1 | 0.002 |
| 39 | 0.160 | 0.164 | 0.145 | 0.049 | 0.085 | 0.033 | 0.4 | 0.004 |
| 40 | 0.135 | 0.143 | 0.111 | 0.441 | 0.506 | 0.389 | 3.4 | 0.001 |
| 41 | 0.109 | 0.143 | 0.101 | 0.493 | 0.691 | 0.411 | 4.5 | 0.008 |
| 43 | 0.134 | 0.137 | 0.132 | 0.255 | 0.223 | 0.268 | 1.9 | 0.001 |
| 52 | 0.315 | 0.306 | 0.503 | 0.745 | 0.734 | 0.782 | 2.0 | 0.005 |
| 53 | 0.365 | 0.363 | 0.464 | 0.169 | 0.236 | 0.256 | 0.6 | 0.014 |
| 58 | 0.136 | 0.151 | 0.173 | 0.257 | 0.299 | 0.353 | 2.0 | 0.007 |
| 59 | 0.043 | 0.035 | 0.043 | 0.020 | 0.000 | 0.000 | 0.2 | 0.008 |
| 61 | 0.048 | 0.049 | 0.067 | 0.029 | 0.018 | 0.000 | 0.4 | 0.040 |
| 63 | 0.192 | 0.175 | 0.155 | 0.405 | 0.398 | 0.460 | 2.4 | 0.000 |
| 67 | 0.000 | 0.000 | 0.000 | 0.119 | 0.155 | 0.153 | A* | 0.003 |
| 70 | 0.179 | 0.243 | 0.183 | 0.349 | 0.364 | 0.461 | 1.9 | 0.010 |
| 72 | 0.000 | 0.000 | 0.000 | 0.160 | 0.035 | 0.124 | A | 0.046 |
| 73 | 0.041 | 0.049 | 0.056 | 0.102 | 0.184 | 0.114 | 2.7 | 0.031 |
| *A: appeared; spot ID, spot numbers shown in Fig. 3; BJBN-1-3, replicates 1 to 3 of hybrids of wild mustard (BJ) with nontransgenic seeds; BJtBN-1-3, replicates 1 to 3 of hybrids of BJ with transgenic oilseed rape seeds; BJtBN/BJBN, normalized spot volume in BJtBN seeds divided by the normalized volume in BJBN seeds. | | | | | | | | |

| **Table S4. Volume (%), ratio and p value of the differentially accumulated protein spots between hybrids of wild mustard (BJ) with nontransgenic (BJBN) and BJ with transgenic (BJtBN) oilseed rape seeds.** | | | | | | | | |
| --- | --- | --- | --- | --- | --- | --- | --- | --- |
| Spot ID | BJ-1 | BJ-2 | BJ-3 | BNBJ-1 | BNBJ-2 | BNBJ-3 | BJBN/BJ | p value |
| 33 | 0.060 | 0.082 | 0.108 | 0.213 | 0.144 | 0.207 | 2.1 | 0.030 |
| 34 | 0.249 | 0.303 | 0.318 | 0.106 | 0.098 | 0.088 | 0.3 | 0.001 |
| 35 | 0.034 | 0.019 | 0.040 | 0.293 | 0.229 | 0.223 | 7.9 | 0.001 |
| 36 | 0.041 | 0.016 | 0.040 | 0.142 | 0.155 | 0.110 | 4.2 | 0.003 |
| 37 | 0.025 | 0.053 | 0.021 | 0.335 | 0.274 | 0.203 | 8.1 | 0.004 |
| 38 | 0.000 | 0.027 | 0.000 | 0.114 | 0.135 | 0.091 | 12.6 | 0.003 |
| 39 | 0.054 | 0.055 | 0.042 | 0.160 | 0.164 | 0.145 | 3.1 | 0.000 |
| 41 | 0.041 | 0.079 | 0.078 | 0.109 | 0.143 | 0.101 | 1.8 | 0.045 |
| 42 | 0.165 | 0.243 | 0.257 | 0.614 | 0.562 | 0.476 | 2.5 | 0.003 |
| 43 | 0.068 | 0.109 | 0.057 | 0.134 | 0.137 | 0.132 | 1.7 | 0.023 |
| 44 | 0.126 | 0.169 | 0.131 | 0.449 | 0.264 | 0.385 | 2.6 | 0.016 |
| 45 | 0.059 | 0.102 | 0.200 | 0.349 | 0.251 | 0.266 | 2.4 | 0.031 |
| 46 | 0.000 | 0.000 | 0.000 | 0.048 | 0.044 | 0.038 | A* | 0.000 |
| 47 | 0.094 | 0.108 | 0.105 | 0.016 | 0.012 | 0.029 | 0.2 | 0.000 |
| 48 | 0.021 | 0.051 | 0.028 | 0.091 | 0.081 | 0.093 | 2.6 | 0.005 |
| 49 | 0.000 | 0.000 | 0.000 | 0.110 | 0.087 | 0.106 | A | 0.000 |
| 50 | 0.101 | 0.141 | 0.126 | 0.092 | 0.059 | 0.041 | 0.5 | 0.037 |
| 51 | 0.000 | 0.000 | 0.000 | 0.093 | 0.080 | 0.097 | A | 0.000 |
| 52 | 1.059 | 1.697 | 1.184 | 0.315 | 0.306 | 0.503 | 0.3 | 0.010 |
| 53 | 0.000 | 0.000 | 0.000 | 0.365 | 0.363 | 0.464 | A | 0.000 |
| 54 | 0.173 | 0.192 | 0.128 | 0.043 | 0.046 | 0.050 | 0.3 | 0.004 |
| 55 | 0.000 | 0.000 | 0.000 | 0.321 | 0.227 | 0.129 | A | 0.015 |
| 56 | 0.158 | 0.137 | 0.183 | 0.039 | 0.077 | 0.060 | 0.4 | 0.004 |
| 57 | 0.056 | 0.069 | 0.063 | 0.101 | 0.102 | 0.111 | 1.7 | 0.001 |
| 59 | 0.086 | 0.115 | 0.078 | 0.043 | 0.035 | 0.043 | 0.4 | 0.010 |
| 60 | 0.104 | 0.101 | 0.103 | 0.022 | 0.021 | 0.029 | 0.2 | 0.000 |
| 61 | 0.134 | 0.106 | 0.104 | 0.048 | 0.049 | 0.067 | 0.5 | 0.006 |
| 62 | 0.205 | 0.194 | 0.246 | 0.126 | 0.121 | 0.104 | 0.5 | 0.005 |
| 63 | 0.131 | 0.128 | 0.074 | 0.192 | 0.175 | 0.155 | 1.6 | 0.042 |
| 64 | 0.720 | 0.753 | 0.696 | 0.210 | 0.146 | 0.249 | 0.3 | 0.000 |
| 65 | 0.186 | 0.155 | 0.198 | 0.876 | 1.132 | 1.250 | 6.0 | 0.001 |
| 66 | 0.253 | 0.483 | 0.315 | 1.656 | 1.453 | 1.543 | 4.4 | 0.000 |
| 68 | 0.430 | 0.534 | 0.392 | 0.676 | 0.661 | 0.721 | 1.5 | 0.007 |
| 69 | 0.376 | 0.319 | 0.336 | 0.111 | 0.122 | 0.139 | 0.4 | 0.000 |
| 70 | 0.074 | 0.110 | 0.117 | 0.179 | 0.243 | 0.183 | 2.0 | 0.015 |
| 71 | 0.010 | 0.016 | 0.000 | 0.048 | 0.063 | 0.079 | 7.5 | 0.006 |
| 73 | 0.021 | 0.029 | 0.017 | 0.041 | 0.049 | 0.056 | 2.2 | 0.009 |
| *A: appeared; spot ID, spot numbers shown in Fig. 4; BJ-1-3, replicates 1 to 3 of wild mustard (BJ) seeds; BJBN-1-3, replicates 1 to 3 of hybrids of BJ with nontransgenic oilseed rape seeds; BJBN/BJ, normalized spot volume in BJBN seeds divided by the normalized volume in BJ seeds. | | | | | | | | |
